# Linezolid MIC creep among clinically significant *S. aureus*: a retrospective eight-year study

**DOI:** 10.3389/frabi.2026.1814348

**Published:** 2026-07-15

**Authors:** Chinchana Shylaja Eshwarappa, Veerabhadra Swamy GS, Supreetha R. Shettar, Yogeesh D. Maheshwarappa, Mahadevaiah Neelambike Sumana, Tejashree A, Rashmi P. Mahale, Sowmya GS, Morubagal Raghavendra Rao, Ranjitha Shankaregowda, Vidyavathi B. Chitaragi, Deepashree R, Sujatha SR, Neetha S. Murthy, Badveti Satya Sai, Vasimalli Vinay Kumar

**Affiliations:** Department of Microbiology, JSS Medical College and Hospital, JSS AHER, Mysuru, India

**Keywords:** antibiotic susceptibility testing (AST), linezolid, minimal inhibition concentration (MIC), MIC creep, methicillin-resistant Staphylococcus aureus (MRSA), methicillinsusceptible staphylococcus aureus (MSSA)

## Abstract

**Introduction:**

The rising threat of antimicrobial resistance, particularly the phenomenon of MIC creep, poses a significant challenge to the clinical effectiveness of key antibiotics. Linezolid is a crucial last-resort agent for treating severe infections caused by Gram-positive pathogens, including methicillin-resistant *Staphylococcus aureus* (MRSA). This study aimed to evaluate the temporal trends of linezolid minimum inhibitory concentrations (MICs) in clinical *S. aureus* isolates at a tertiary care hospital over an eight-year period.

**Methods:**

A retrospective study was conducted on clinical *S. aureus* isolates over eight years from 2017–2024 in a tertiary care hospital. MICs were determined using the VITEK-2 system and interpreted according to CLSI guidelines. Data were analyzed using IBM SPSS Statistics version 20.0.

**Results:**

From 2017 to 2024, A total of 4,325 *S. aureus* isolates were analyzed, comprising 2,682 (62.01%) MSSA and 1,643 (37.98%) MRSA. The mean linezolid MIC for MSSA increased from 1.41 ± 0.53 µg/mL (2017) to 1.72 ± 0.45 µg/mL (2024), and for MRSA from 1.34 ± 0.47 µg/mL (2017) to 1.78 ± 0.42 µg/mL (2024). A progressive rightward shift in MIC distribution from 1 µg/mL to 2 µg/mL was observed in both groups. Spearman’s rank correlation demonstrated a significant positive trend (MSSA: *r_s_* = 0.893, *p* = 0.001; MRSA: *r_s_* = 0.857, *p* = 0.001), consistent with the “MIC creep.” All isolates remained within the CLSI susceptibility breakpoint (≤ 4 µg/mL), and no high-level resistance was detected.

**Conclusion:**

Linezolid continues to be highly effective; however, the progressive MIC creep from 1 µg/mL to 2 µg/mL indicates emerging reduced susceptibility. These findings stress the need for ongoing MIC surveillance and strict antibiotic stewardship to preserve linezolid efficacy.

## Introduction

*Staphylococcus aureus* causes a wide range of infections, including bacteremia, endocarditis, pneumonia, osteoarticular infections, and skin and soft tissue infections. Methicillin-resistant *Staphylococcus aureus* (MRSA) was first identified in the United Kingdom in 1961 (Jevons, 1961). Since then, MRSA has disseminated worldwide, becoming a significant public health issue because of its role in difficult-to-treat and costly hospital-acquired infections (Hsieh et al., 2016).

Most MRSA strains remain susceptible only to vancomycin, which has been extensively used for their treatment. This raises concern that MRSA could act as a reservoir for the emergence of vancomycin-resistant strains that are untreatable with existing antibiotics (Matuszewska et al., 2020). For nearly two decades, vancomycin has been considered the drug of choice for MRSA infections. However, recent reports of therapeutic failure with vancomycin in MRSA cases have heightened concerns over strains for which no effective therapy might remain (Roch et al., 2014).

Several hospital-associated MRSA clones have acquired multidrug resistance and exhibit decreased susceptibility to vancomycin. Although vancomycin remains the first-line intravenous treatment for severe MRSA infections, vancomycin-resistant (*VRSA*) and vancomycin-intermediate *S. aureus* (*VISA*) isolates are increasingly reported worldwide. In such cases, linezolid is considered a critical life-saving therapeutic option (Holden et al., 2004; Peer et al., 2011). Linezolid, the first oxazolidinone antimicrobial introduced in 2000, is the only oral antibiotic available for resistant *Staphylococcus* infections (Zahedi Bialvaei et al., 2017).

It has demonstrated efficacy against skin and soft tissue infections, nosocomial pneumonia (including ventilator-associated pneumonia), infective endocarditis, and MRSA meningitis. Moreover, linezolid has been effective in eradicating MRSA colonization from the nose and throat. Its mechanism of action involves inhibition of bacterial protein synthesis by binding to the peptidyl transferase center (PTC) of the 50S ribosomal subunit (Venkata et al., 2014).

To date, reported mechanisms of linezolid resistance in clinical *S. aureus* isolates include: (i) mutations in the domain V region of one or more copies of the 23S rRNA gene (Zahedi Bialvaei et al., 2017); (ii) acquisition of the plasmid-mediated ribosomal methyltransferase cfr gene; and (iii) deletions or mutations in ribosomal protein L3 of the PTC. Additional domain V mutations in the 23S rRNA and substitutions in ribosomal protein L4 have also been described in laboratory-derived linezolid-resistant *S. aureus* strains (Venkata et al., 2014).

Linezolid resistance was first reported within a year of its clinical approval. Although uncommon, resistant *S. aureus* isolates have been described from different regions worldwide. In India, the earliest report of linezolid resistance was published in 2011 from Kashmir (Sharlee and Sumangala, 2020).

The objective of the present study was to evaluate the trends in linezolid minimum inhibitory concentrations (MICs) among clinically significant *S. aureus* isolates from a tertiary care hospital over a period of eight years.

## Materials and methods

### Sample/data collection

This retrospective study was conducted using culture and antimicrobial susceptibility testing (AST) data collected from clinically significant Methicillin-resistant *Staphylococcus aureus* (MSSA and MRSA) isolates obtained from various clinical specimens processed at the Clinical Microbiology Laboratory of a tertiary care 1,800-bed hospital in Mysuru, South India. The study period spanned eight years, from January 2017 to December 2024. Data were retrieved from the hospital’s Health Information System (HIS) and included patient demographics, ward, specimen type, and microbiological results. Clinical specimens included pus, blood, cerebrospinal fluid (CSF), ear swabs, urine, vaginal swabs, and other relevant samples submitted for diagnostic purposes.

### Inclusion and exclusion criteria

All *Staphylococcus aureus* isolates recovered from clinical specimens submitted for routine diagnostic purposes at the study center between 2017 and 2024 were included in the analysis. Only isolates reported as clinically significant based on standard microbiological laboratory criteria and clinical relevance were considered.

Likely contaminants or colonizing isolates were excluded based on specimen type, culture characteristics, and routine laboratory reporting practices. As the study was designed as an isolate-level analysis, multiple isolates from the same patient were included, and Duplicate isolates from the same patient were included to comprehensively assess temporal MIC trends. No restriction to a first isolate per patient was applied, and all eligible isolates during the study period were analyzed to comprehensively assess temporal trends in linezolid minimum inhibitory concentrations (MICs).

### Sample processing

All samples were processed according to standard microbiological procedures. Upon receipt in the microbiology laboratory, specimens were subjected to direct microscopy (Gram staining or wet mount, as applicable), followed by inoculation on appropriate culture media- media depending on the specimen type. Cultures were incubated aerobically at 37 °C for 24–48 hours.

Growth from cultures was evaluated, and colonies suspected to be *Staphylococcus aureus* were identified using conventional and confirmed by automated identification using the VITEK 2 Compact system (bioMérieux, France). Methicillin resistance was determined by cefoxitin screening and automated susceptibility results. Antimicrobial susceptibility testing, including determination of the Minimum Inhibitory Concentration (MIC) for linezolid was collected from VITEK 2 (GP AST-P628 card). MIC values were interpreted and reported as Susceptible (S), Intermediate (I), or Resistant (R) according to the Clinical and Laboratory Standards Institute (CLSI) guidelines.

### Ethical considerations

Our study involved the analysis of anonymized, laboratory-generated data obtained from routine clinical diagnostics and did not involve direct patient participation. The Institutional Ethics Committee of JSS Medical College, Mysuru, reviewed the study protocol and confirmed exemption from ethical approval.

### Statistical analysis

Descriptive statistics were used to evaluate trends in linezolid MIC values and resistance patterns among MRSA isolates. Year-wise comparisons were made for demographic characteristics, specimen distribution, and MIC data. Chi-square tests were used to assess associations between variables, with p-values <0.0001 considered statistically significant. Data were analyzed using IBM SPSS Statistics version 20.0.

## Results

A total of 4,325 *Staphylococcus aureus* isolates were recovered and analyzed over an eight-year study period (2017–2024). The cumulative distribution of phenotypes was comprised of 62.01% MSSA (n = 2,682) and 37.99% MRSA (n = 1,643) (**Table 1**).

**Table 1:** Annual distribution and methicillin resistance patterns of *S. aureus* isolates (2017–2024).

Year	Total isolates (n)	MSSA n (%)	MRSA n (%)	χ^2^	p-value
2017	428	265 (61.9)	163 (38.1)	—	(Baseline)
2018	561	348 (62.0)	213 (38.0)	0.001	0.970
2019	612	379 (61.9)	233 (38.1)	0.000	0.996
2020	409	254 (62.1)	155 (37.9)	0.003	0.955
2021	475	295 (62.1)	180 (37.9)	0.003	0.953
2022	595	369 (62.0)	226 (38.0)	0.001	0.973
2023	613	380 (62.0)	233 (38.0)	0.001	0.980
2024	632	392 (62.0)	240 (38.0)	0.001	0.971
Total	**4,325**	2,682(62.01)	1,643(37.99)	**0.007**	**0.84**

Bold values indicate statistically significant findings (p < 0.05).

The annual distribution of both MSSA and MRSA remained remarkably stable throughout the study. In 2017, the proportions were 61.92% MSSA and 38.08% MRSA. By the end of the study period in 2024, these figures were nearly identical, at 62.03% MSSA and 37.97% MRSA. Chronologically, the annual isolation rate for MSSA fluctuated only slightly, with a peak of 62.11% in 2021, while the MRSA rate peaked at 38.08% in 2017.

Statistical analysis using the Pearson’s Chi-square test of independence was performed to evaluate the relationship between the isolation year and the distribution of these two phenotypes. The results confirmed no significant variation in the proportions of MSSA and MRSA over the eight-year period (*X^2^* = 0.007, p = 0.84). This indicates that the ratio of susceptible to resistant *S. aureus* has maintained a consistent baseline, with no significant trend toward increased resistance or increased susceptibility in the study population.

The distribution of isolates varied significantly by age and gender. Males represented 62.9% (n=2,722) of total cases, with an overall M:F ratio of 1.69. The highest burden was in the 21–60 age group.

Statistical testing showed a significant association between gender and phenotype (p = 0.008) and age and phenotype (p = 0.019). Interestingly, in the 21–30 age group, the M:F ratio for MRSA dropped to 0.59, showing a female predominance. However, in the Geriatric group (>60 years), the M:F ratio increased to 2.59 for MRSA, showing that older males are at much higher risk (**Table 2**). Furthermore, the distribution of isolates varied according to specimen type and hospital location (**Table 3**). In both groups, the majority of isolates were obtained from pus samples (MSSA: 48%, n = 1,287; MRSA: 47.3%, n = 777), followed by respiratory samples (MSSA: 30%, n = 805; MRSA: 23%, n = 378) and blood (MSSA: 14%, n = 375; MRSA: 18%, n = 296). Urine and other sample types contributed to a smaller proportion of isolates in both MSSA and MRSA groups. With respect to hospital location, a significantly higher proportion of MRSA isolates were recovered from intensive care units (22%, n = 361) compared to MSSA (5%, n = 134), whereas the majority of MSSA isolates originated from general wards (95%, n = 2,548). This finding suggests a greater burden of MRSA in critical care settings. Notably, a targeted analysis of these ICU-derived isolates revealed that between 65% and 69% exhibited a linezolid MIC of 2 µg/mL. These findings suggest that critical care settings, characterized by high antimicrobial pressure, may be the primary focal points for the observed population-level MIC creep.

**Table 2:** Demographic distribution and M:F ratios.

Age Category	MSSA (M/F)	M:F Ratio (MSSA)	MRSA (M/F)	M:F Ratio (MRSA)	p-value
Pediatric (0–20)	362/192	1.88	171/121	1.41	**0.019 (Age)** **0.008 (Sex)**
Adult (21–60)	968/559	1.73	589/439	1.34	
Geriatric (>60)	399/202	1.97	233/90	2.59	
Total	**1,729/953**	**1.81**	**993/650**	**1.53**	

Bold values indicate statistically significant findings (p < 0.05).

**Table 3:** Distribution of MSSA and MRSA isolates by sample type and location.

Category	Subcategory	MSSA (n=2,682) n (%)	MRSA (n=1,643) n (%)
Sample Type	Pus	1,287 (48%)	777 (47.3%)
	Respiratory	805 (30%)	378 (23%)
	Blood	375 (14%)	296 (18%)
	Urine	80 (3%)	115 (7%)
	Other	135 (5%)	77 (4.7%)
Location	ICU	134 (5%)	361 (22%)
	Wards	2,548 (95%)	1,282 (78%)
Total	—	**2,682 (100%)**	**1,643 (100%)**

Bold values indicate statistically significant findings (p < 0.05).

### Linezolid MIC distribution patterns

Across the eight-year period, 4,325 *S. aureus* isolates were tested for linezolid minimum inhibitory concentration (MIC). The majority (2,766 [63.95%]) exhibited an MIC of 2 µg/mL, followed by 1,502 [34.73%] with an MIC of 1 µg/mL. MIC values of ≤ 0.5 µg/mL, 4 µg/mL, and ≥ 8 µg/mL were rare (0.37%, 0.74%, and 0.02%, respectively), indicating sustained linezolid susceptibility with only isolated higher MIC observations (**Table 4**).

**Table 4:** Year-wise distribution of linezolid MIC values among *S. aureus* isolates (2017–2024).

Year (n)	≤ 0.5 (n, %)	1 (n, %)	2 (n, %)	4 (n, %)	≥ 8 (n, %)
2017 (428)	7 (1.64)	257 (60.05)	152 (35.51)	4 (0.93)	0 (0.00)
2018 (561)	0 (0.00)	256 (45.63)	294 (52.40)	10 (1.78)	1 (0.18)
2019 (612)	2 (0.33)	223 (36.44)	385 (62.91)	2 (0.33)	0 (0.00)
2020 (409)	3 (0.73)	186 (45.48)	215 (52.57)	5 (1.22)	0 (0.00)
2021 (475)	0 (0.00)	119 (25.05)	354 (74.53)	2 (0.42)	0 (0.00)
2022 (595)	3 (0.50)	133 (22.35)	453 (76.13)	6 (1.01)	0 (0.00)
2023 (613)	0 (0.00)	164 (26.76)	449 (73.24)	0 (0.00)	0 (0.00)
2024 (632)	1 (0.16)	164 (25.95)	464 (73.42)	3 (0.47)	0 (0.00)
**Total (4,325)**	**16 (0.37)**	**1,502 (34.73)**	**2,766 (63.95)**	**32 (0.74)**	**1 (0.02)**

Bold values indicate statistically significant findings (p < 0.05).

### Temporal redistribution and “MIC Creep” of linezolid

**Figure 1:**
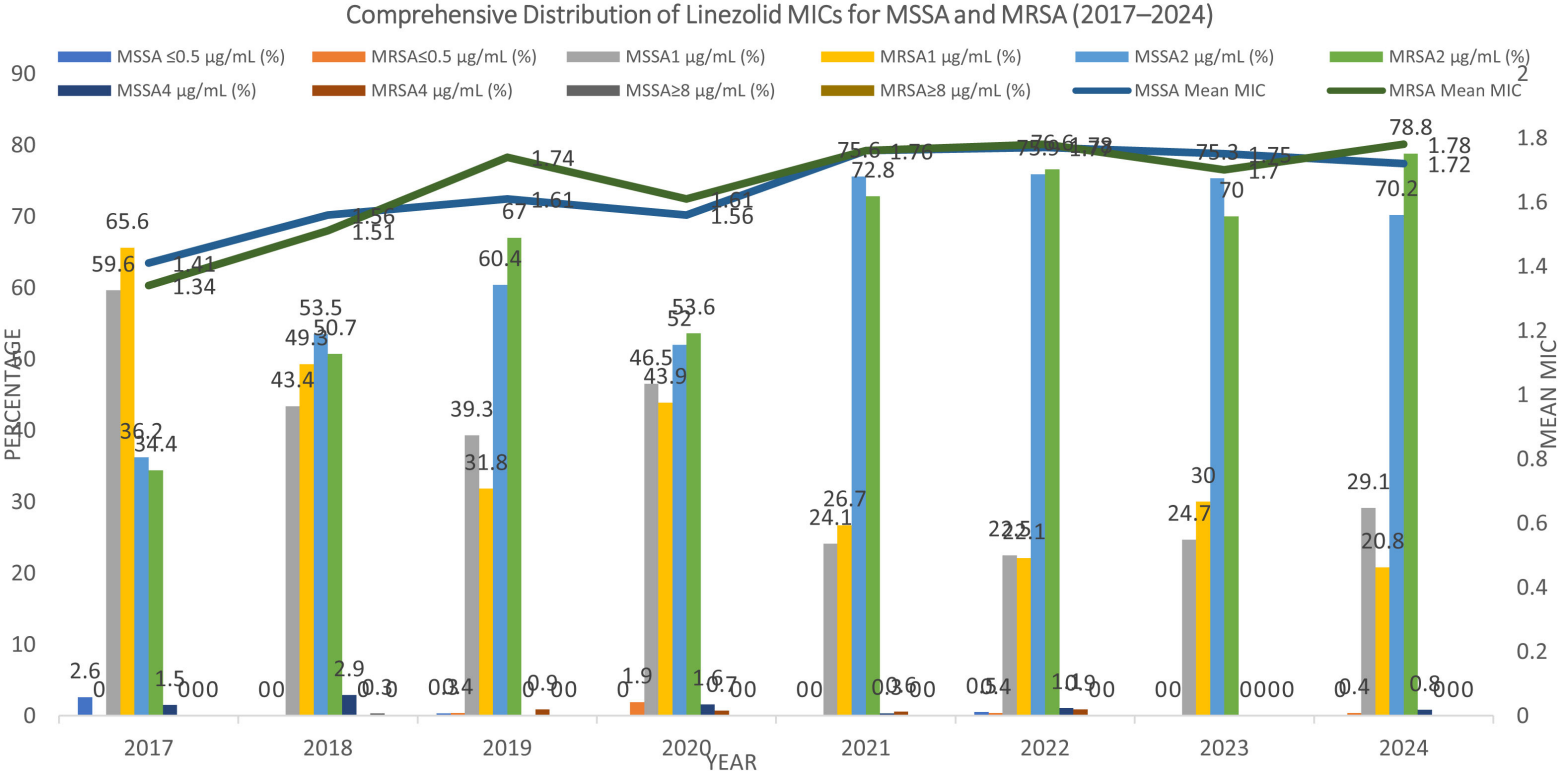
Comprehensive distribution of linezolid MICs for MSSA and MRSA (2017–2024).

A comprehensive analysis of linezolid Minimum Inhibitory Concentrations (MIC) over the eight-year study period revealed a significant longitudinal MIC creep among both MSSA and MRSA isolates (**Table 5**, **Figure 1**). While the population-level susceptibility was maintained within CLSI limits, a distinct shift in the distribution was observed; in 2017, the MIC_50_ was 1 µg/mL for both phenotypes, but it increased to 2 µg/mL in 2018 and remained stable at that level through 2024. Notably, the MIC_90_ remained constant at 2 µg/mL throughout the study.

**Table 5:** Comprehensive distribution of linezolid MICs for MSSA and MRSA (2017–2024).

Year	Phenotype (n)	≤0.5 µg/mL n(%)	1 µg/mL n(%)	2 µg/mL n(%)	4 µg/mL n(%)	≥8 µg/mL n(%)	MIC_50_	MIC_90_	Mean MIC ± SD
2017	MSSA (265)	7 (2.6)	158 (59.6)	96 (36.2)	4 (1.5)	0 (0.0)	1	2	1.41 ± 0.53
	MRSA (163)	0 (0.0)	107 (65.6)	56 (34.4)	0 (0.0)	0 (0.0)	1	2	1.34 ± 0.47
2018	MSSA (348)	0 (0.0)	151 (43.4)	186 (53.5)	10 (2.9)	1 (0.3)	2	2	1.56 ± 0.59
	MRSA (213)	0 (0.0)	105 (49.3)	108 (50.7)	0 (0.0)	0 (0.0)	2	2	1.51 ± 0.50
2019	MSSA (379)	1 (0.3)	149 (39.3)	229 (60.4)	0 (0.0)	0 (0.0)	2	2	1.61 ± 0.48
	MRSA (233)	1 (0.4)	74 (31.8)	156 (67.0)	2 (0.9)	0 (0.0)	2	2	1.74 ± 0.52
2020	MSSA (254)	0 (0.0)	118 (46.5)	132 (52.0)	4 (1.6)	0 (0.0)	2	2	1.56 ± 0.55
	MRSA (155)	3 (1.9)	68 (43.9)	83 (53.6)	1 (0.7)	0 (0.0)	2	2	1.61 ± 0.57
2021	MSSA (295)	0 (0.0)	71 (24.1)	223 (75.6)	1 (0.3)	0 (0.0)	2	2	1.76 ± 0.46
	MRSA (180)	0 (0.0)	48 (26.7)	131 (72.8)	1 (0.6)	0 (0.0)	2	2	1.76 ± 0.45
2022	MSSA (369)	2 (0.5)	83 (22.5)	280 (75.9)	4 (1.1)	0 (0.0)	2	2	1.77 ± 0.49
	MRSA (226)	1 (0.4)	50 (22.1)	173 (76.6)	2 (0.9)	0 (0.0)	2	2	1.78 ± 0.48
2023	MSSA (380)	0 (0.0)	94 (24.7)	286 (75.3)	0 (0.0)	0 (0.0)	2	2	1.75 ± 0.44
	MRSA (233)	0 (0.0)	70 (30.0)	163 (70.0)	0 (0.0)	0 (0.0)	2	2	1.70 ± 0.46
2024	MSSA (392)	0 (0.0)	114 (29.1)	275 (70.2)	3 (0.8)	0 (0.0)	2	2	1.72 ± 0.45
	MRSA (240)	1 (0.4)	50 (20.8)	189 (78.8)	0 (0.0)	0 (0.0)	2	2	1.78 ± 0.42

Linear trend analysis confirmed a statistically significant decline in the proportion of isolates with MICs ≤1 µg/mL (p < 0.001) and a concomitant increase in isolates with an MIC of 2 µg/mL (p < 0.001) (**Table 6**). This upward shift was further validated by Spearman’s correlation coefficients (MSSA r_s_= 0.893; MRSA r_s_ = 0.857), indicating a strong monotonic trend toward higher MIC values over time (**Table 7**). Despite an increase in the mean MIC (+0.31 µg/mL for MSSA and +0.44 µg/mL for MRSA), no significant trend was observed for MIC values ≥4 µg/mL, and no linezolid-resistant strains were detected in the final six years of the study.

**Table 6:** Linear trend analysis of linezolid MIC categories (This table demonstrates the statistical significance of the population shift for each MIC value.) .

MIC (µg/mL)	Phenotype	χ^2^ (linear trend)	p-value	Direction	Interpretation
≤ 1	MSSA/MRSA	38.90/44.32	< 0.001	**Decreasing**	Significant loss of highly susceptible isolates
2	MSSA/MRSA	41.72/47.61	< 0.001	**Increasing**	Evidence of significant “MIC Creep”
≥ 4	MSSA/MRSA	0.91/0.62	0.34/0.43	**Stable**	No significant emergence of resistance

Bold values indicate statistically significant findings (p < 0.05).

**Table 7:** Correlation and monotonic trend summary.

Parameter	MSSA value	MRSA value	Significance
Spearman’s rs	0.893	0.857	Strong positive monotonic trend (p < 0.001)
Mean MIC Shift	+0.31 µg/mL	+0.44 µg/mL	Greater increase observed in MRSA
MIC_50_ Shift	1 → 2 µg/mL	1 → 2 µg/mL	Population-level baseline creep
CLSI Status	Susceptible	Susceptible	No isolates exceeded resistance breakpoints

## Discussion

*Staphylococcus aureus* remains one of the most significant human pathogens, responsible for a wide spectrum of hospital- and community-acquired infections such as pneumonia, surgical site infections, and bacteremia. The global emergence of multidrug-resistant strains, particularly methicillin-resistant *S. aureus* (MRSA), continues to challenge clinicians and microbiologists in infection control and therapeutic management (Kale and Patil, 2019). This eight-year retrospective study provides longitudinal insights into the evolving linezolid susceptibility patterns at a tertiary care center in South India.

In the present study, methicillin-sensitive *S. aureus* (MSSA) was more prevalent 2,682 (62.01%) than MRSA 1,643 (37.98%). This pattern closely mirrors other South Indian reports, such as those by Joshi et al. (2013), Khadri and Alzohairy, (2010), and Pai et al. (2010), which recorded MRSA rates between 36% and 38% (Joshi et al., 2013; Khadri and Alzohairy, 2010; Arumugam et al., 2023). The Indian Network for Surveillance of Antimicrobial Resistance (INSAR, 2025) and Arora et al. (2020) reported slightly higher MRSA rates in North India (41–46%) (Patil et al., 2022; Sharma et al., 2024). Globally, MRSA prevalence is lower, averaging 14.7%, with the highest rates reported in Asian countries compared with Europe and North America (Makeri et al., 2023). These findings indicate that while MRSA remains a significant clinical concern, MSSA continues to constitute the majority of *S. aureus* isolates both regionally and worldwide.

A male predominance was observed, accounting for 62.93% of infections, which aligns with previous reports by Pavani Chitthaluru et al. (2024) and Sharlee et al. (2020), who recorded higher proportions of MRSA among males (67.7% vs. 32.3% and 62% vs. 38%, respectively) (Sharlee and Sumangala, 2020; Chitthaluru et al., 2024). Similarly, Nadia Aslam et al. (2013) reported a higher prevalence of MRSA among males (65.2%) than females (34.8%) (Aslam et al., 2013). Interestingly, we noted a female predominance in the 21–30-year age group, a finding that contrasts with most international literature but has been documented in specific studies from North India and Africa (Ike et al., 2016; Anucha et al., 2021; Bunza et al., 2019) (Humphreys et al., 2015; Sarrafzadeh et al., 2018). However, similar findings were documented by Bunza et al. (2019) from North India and by Ike B et al. (2016) and Anucha et al. (2021) from Africa, where female prevalence ranged from 58% to 62% (Ike et al., 2016; Anucha et al., 2021; Bunza et al., 2019). These variations suggest that gender-related differences in *S. aureus* infections may be influenced by regional demographic, hormonal, occupational, or behavioral factors (Ager and Gould, 2012).

Since its clinical introduction in 2000, linezolid has remained a vital oxazolidinone for treating multidrug-resistant Gram-positive pathogens, specifically targeting methicillin-resistant *Staphylococcus aureus* (MRSA) and vancomycin-resistant *Enterococcus* (VRE) (Thirot et al., 2024) (Belzile and Bunce, 2025). Its near-complete oral bioavailability and high tissue penetration facilitate a seamless transition from intravenous to oral therapy, making it a mainstay for managing complex skin and soft-tissue infections as well as drug-resistant tuberculosis (Bunce, 2025) (Hui et al., 2024). Its oral bioavailability and favorable pharmacokinetics make it a cornerstone for both inpatient and outpatient therapy and is primarily driven by the (AUC_0–24_/MIC) ratio; however, recent evidence emphasizes that maintaining this ratio within a specific therapeutic window is critical to prevent hematological toxicities (Hui et al., 2024) (Thirot et al., 2024). Consequently, even subtle shifts in MIC within the susceptible range may affect pharmacodynamic efficacy and clinical outcomes, emphasizing the importance of continuous MIC surveillance.

Analysis of MIC distributions revealed a significant “rightward shift” over time. Isolates with an MIC of 2 µg/mL increased from 35.51% in 2017 to 73.42% in 2024, while those with an MIC of 1 µg/mL declined from 60.05% to 25.95%. The mean MIC also showed a statistically significant positive correlation with time (r_s_ = 0.893, p < 0.001), confirming a gradual “MIC creep” phenomenon within the susceptible population. This observation is consistent with earlier Indian studies. Rajan et al. (2017) reported an increasing proportion of *S. aureus* isolates with MIC ≥ 2 µg/mL in South India (Rajan et al., 2017), while Kumar et al. (2017) observed a modest rise in the geometric mean MIC (2.20 to 2.29 µg/mL) with over 40% of isolates reaching MIC 3 µg/mL (Kumari et al., 2019). Such creeping shifts within the susceptible range warrant close attention, as they may precede clinical resistance emergence.

Comparable findings have been documented internationally. Miyazaki et al. (2014) reported a rise in geometric mean MIC for MRSA from 1.178 to 1.582 µg/mL over five years in Japan (Miyazaki et al., 2014). Similarly, Livermore et al. (2020) noted a linezolid MIC creep among *S. aureus* isolates in China over a decade, although variations between MRSA and MSSA were minimal, suggesting clonal or regional influences (Livermore et al., 2011). According to the 20-year SENTRY Antimicrobial Surveillance Program report (1997 to 2016), the global linezolid MIC_90_ and modal MIC for *S. aureus* and coagulase-negative staphylococci have remained stable at 2 and 1 µg/mL, respectively, with susceptibility rates consistently exceeding 99% (Diekema et al., 2019). Taken together, our findings corroborate these global patterns, demonstrating that while clinical resistance remains rare, the gradual upward shift in MIC values signifies a measurable decline in susceptibility at the population level.

In our study, the majority of *S. aureus* isolates remained susceptible to linezolid, with only a small fraction exhibiting resistance at MIC = 4 µg/mL and no high-level resistance (MIC ≥ 8 µg/mL) except for a single MSSA isolate. This finding echoes the surveillance data from Miyazaki et al. (2014) in Japan, where no isolates exceeded MIC 8 µg/mL over five years, underscoring the sustained efficacy of linezolid (Miyazaki et al., 2014). While early studies frequently reported complete susceptibility, more recent investigations have identified both MRSA and MSSA strains resistant to linezolid, often linked to the presence of the cfr gene or mutations in domain V of the 23S rRNA gene (Shore et al., 2010). Because molecular characterization was not performed in the present study, the specific mechanisms underlying the observed MIC creep remain undetermined. However, based on previously published literature, such trends are often associated with the acquisition of the plasmid-mediated *cfr* gene or specific ribosomal mutations (e.g., G2576T) (Rajan et al., 2014; Zhou et al., 2025). While our discussion of these mechanisms is speculative regarding our specific isolates, the rarity of high-level resistance in our dataset—contrasted with the documented emergence of *cfr*-mediated resistance elsewhere in India—highlights the urgent need for future molecular surveillance to preserve linezolid’s effectiveness. This aligns with the study conducted by Shan Zhou et al. (2025) (Zhou et al., 2025) in Japan showed out of 797 *S. aureus* isolates, eight exhibited elevated linezolid MIC ≥8μg/mL according to VITEK2 AST-GP67 results. However, only one isolate has been confirmed as linezolid-resistant (MIC 8μg/mL). Where Linezolid resistance-associated genes, including transferable element genes (*cfr, cfr(B), optrA, poxtA*) and ribosomal mutations (23S rRNA and L3, L4, L22), were analyzed in the eight isolates. None of the genes were detected except for G2576T mutations among two isolates with MICs of 4 μg/mL and 8 μg/mL by BMD, respectively. In the Indian context, Rajan et al. (2014) documented a *S. haemolyticus* isolate harboring both the *cfr* gene and a G2576T mutation in the 23S rRNA, demonstrating a concerning convergence of mobile and mutational resistance mechanisms (Rajan et al., 2014), while Kumari et al. (2019) reported G2447U and C2534U 23S rRNA mutations in linezolid-resistant *S. haemolyticus* isolates from North India (Kumari et al., 2019). The plasmid-mediated *cfr* gene poses a heightened threat given its mobility and potential for horizontal transfer across *staphylococcal species* (Chaudhary et al., 2023).

Strengths of the study: The retrospective study reveals the results of a large number of isolates collected over eight years, which is a potential strength of the study.

Limitations of the study: This study did not include molecular analysis of resistance mechanisms or patient-level clinical outcome data (such as treatment response, mortality, or therapeutic failure), thereby limiting the ability to assess the clinical impact of rising linezolid MICs. As a result, the observed MIC trends should be interpreted as microbiological findings, and their direct implications for treatment outcomes cannot be determined. Additionally, potential data entry errors and the absence of detailed specimen-specific analysis further limit the depth of conclusions drawn. Additionally, as a retrospective analysis based on hospital information system (HIS) data, the study is subject to potential selection bias and data entry errors. To minimize these issues, data were extracted from laboratory records and cross-verified for completeness and consistency, and incomplete or inconsistent entries were excluded wherever applicable. However, residual bias cannot be entirely excluded. The absence of detailed specimen-specific analysis further limits the depth of conclusions drawn.

## Conclusion

Over an eight-year surveillance period, linezolid demonstrated excellent *in-vitro* activity against both methicillin-sensitive and methicillin-resistant *Staphylococcus aureus* isolates at our tertiary care center, with most isolates showing MIC values of 1–2 µg/mL and high-level resistance (MIC ≥ 8 µg/mL) remaining extremely rare. However, a subtle but consistent upward shift in MIC distribution was noted, reflected by an increase in isolates with MIC 2 µg/mL and a corresponding decline in those with MIC 1 µg/mL, suggesting the possibility of linezolid MIC creep. While these values remain within the susceptible range, such may indicate a gradual reduction in susceptibility at the microbiological level. Although the potential pharmacodynamic implications of increasing MICs are recognized, the clinical impact of this trend could not be assessed in the present study due to the absence of patient outcome data. Overall, linezolid continues to represent a cornerstone therapy for multidrug-resistant Gram-positive infections; nevertheless, the observed trend of MIC elevation underscores the importance of ongoing surveillance, judicious antimicrobial use, and further studies incorporating clinical and molecular data to better understand its implications.

## Data Availability

The raw data supporting the conclusions of this article will be made available by the authors, without undue reservation.
